# The “Spider Web” Technique in Difficult Chest Wall Reconstructions: A 5-Year Experience

**DOI:** 10.3390/jcm14092903

**Published:** 2025-04-23

**Authors:** Emanuel Palade, Stefanie Schierholz, Tobias Keck, David Benjamin Ellebrecht

**Affiliations:** 1 Department of Surgery, Iuliu Hatieganu University of Medicine and Pharmacy, 400000 Cluj-Napoca, Romania; 2 Thoracic Surgery Clinic, Leon Daniello Clinical Hospital of Pneumology, 400371 Cluj-Napoca, Romania; 3 Department of Surgery, University Medical Centre Schleswig-Holstein, Campus Luebeck, Ratzeburger Allee 160, 23538 Luebeck, Germany; 4 Department of Thoracic Surgery, Clinic for Pulmonary Diseases Grosshansdorf, 22927 Grosshansdorf, Germany

**Keywords:** spider web technique, chest wall resection, chest wall reconstruction

## Abstract

**Background/Objectives**: Primary chest wall tumors or malignancies of adjacent organs with chest wall infiltration present a significant challenge for surgical resection and reconstruction. Larger defects involving the sternum, resections in the area of the thoracic apertures, or those near the spine are difficult to reconstruct. The reconstruction has to ensure stability, to prevent paradoxical movements and lung herniation, while also achieving a satisfactory cosmetic result. The “spider web” technique restores chest wall stability by creating a web-like framework made of non-resorbable threads fixed to adjacent bony structures. Additionally, a synthetic mesh is placed over the web construct, and both layers are covered with muscles (local muscles or different types of flaps). In this prospective study, clinical data from patients who underwent surgery using the “spider web” technique were analyzed with respect to chest wall stability, procedure-specific complications, pulmonary function, and patient satisfaction. **Methods**: A total of 16 patients receiving 18 chest wall resections and reconstructions using the “spider web” technique were followed for at least one year. Chest wall stability and lung function (FEV1 and DLCO) were assessed. Quality of life, cosmetic satisfaction, potential functional impairment, and analgesic consumption were measured using a modified EORTC QLQ-C30 questionnaire. **Results**: The follow-up period ranged from 12 to 32 months. In all cases, optimal chest wall stability was maintained without impairment of respiratory mechanics. Procedure-specific complications occurred in five cases (27.8%), including seroma (one case), hematoma (two cases), necrosis at the TRAM flap donor site (one case), and mesh infection (one case), all of which were resolved without further complications. Postoperative FEV1 and DLCO were not significantly reduced compared with preoperative values. The global health status score for quality of life was 60 ± 27 points. Nine patients reported being able to ascend at least one floor of stairs without shortness of breath and half of the patients were able to participate in sports activities. One patient required prolonged analgesic medication due to chronic pain. In all cases, patients were satisfied with the cosmetic result. Both 30-day and 90-day mortality were 0%. No local recurrence at the chest wall reconstruction site occurred. **Conclusions**: The “spider web” technique is a highly suitable method for chest wall reconstruction, allowing covering all types of chest wall defects, regardless of size and location. This cost-effective technique not only provides optimal stability but also good functional results.

## 1. Introduction

The integrity and stability of the chest wall are responsible for protecting the intrathoracic organs as well as for ensuring unimpaired pulmonary function due to paradoxical respiratory movements. Chest wall resections and reconstructions are required for a wide range of malignant and non-malignant diseases. Lung cancers with chest wall invasion, primary chest wall tumors, as well as wounds after radiotherapy or infections are the most common causes necessitating chest wall resection and reconstruction [1]. Chest wall reconstruction has long posed a significant challenge for surgeons. Generally, a defect larger than 5 cm in diameter or involving at least two ribs is considered an indication of chest wall reconstruction [1]. Particularly challenging are reconstructions of larger defects involving the sternum, the thoracic apertures (superior and inferior), or resections close to the spine. In such cases, one of the four defect margins corresponds to an anatomical structure that precludes proper fixation of prosthetic material.

A recent review by Girotti PMC and Bianchi F (2023) [1] explored the materials used for chest wall reconstruction focusing on aspects such as chest wall stability, protection of intrathoracic organs, preservation of muscle function, risk of infection, and customization. A wide range of materials have been employed over time, including soft synthetic materials (e.g., polypropylene, polytetrafluoroethylene, nylon, polyglactin, silastic, and silicone meshes), rigid materials (e.g., polymethylmethacrylate, titanium, and steel bars), and biological prostheses (e.g., bovine pericardium, porcine dermis, cadaveric human dermis, and bones). Each of these materials has its own set of advantages and limitations, and the multitude of proposed solutions over time suggests that no material is ideal [1].

In this regard, McCormack et al. and Rudolphy et al. presented the characteristics of the ideal reconstruction material for chest wall reconstruction in their studies published in 1989 and 1991, respectively [2,3]. From their perspective, the characteristics of the ideal reconstruction material are availability, ease of use, adaptability to various sizes and shapes, durability, inertness to body fluids, resistance to infection, translucency to x-rays, incorporation by body tissues, malleable to contours, and to not cause excessive adhesions to the subjacent lung.

In addition to reconstructing the solid component of the chest wall, it is essential to cover the prosthetic material and reconstruct the soft tissues using adjacent musculature (if available) or various types of muscle flaps.

The “spider web” technique was developed to overcome some of the major limitations of other reconstruction methods. This technique is inspired by the natural structure of spider webs, which not only exhibit high strength and a uniform stress distribution but also retain stability even if one or more anchor points of the web loosen [4,5]. The study aims to present the “spider web” technique as a useful method for chest wall reconstruction and to report on the outcomes of its application. The novelty of the study lies in introducing a cost-effective, easy-to-perform, and universally applicable reconstruction technique for chest wall defects.

## 2. Materials and Methods

### 2.1. Collective Description and Parameters Analyzed

Over a 5-year period, a total of 18 chest wall reconstructions using the “spider web” technique were performed on 16 patients (10 male and 6 female). In two patients, two reconstructions were performed on the same site, with the second procedure occurring more than one year after the initial surgery. Data regarding the indication for chest wall resection, number of ribs resected, surface area of the resulting defect, material used for web formation, type of soft tissue reconstruction, length of hospital stay, technique-specific complications, and parameters related to quality of life were prospectively collected.

### 2.2. “Spider Web” Technique

**Figure 1:**
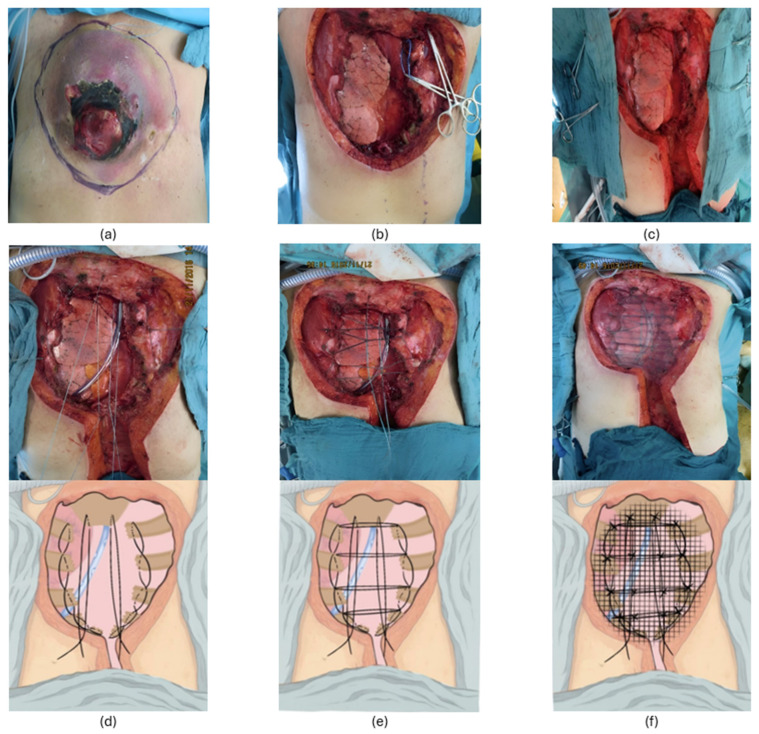
Extended oncologic chest wall resection and reconstruction using the “spider web” technique. (**a**) sternal recurrence of a breast phylloides tumor (cystosarcoma phylloides) after bilateral radical mastectomy; (**b**) large anterior chest wall defect after tumor resection including the entire corpus sterni; (**c**) first step of the reconstruction by placing the two longitudinal axes; (**d**) additional longitudinal wires positioned at the level of the former sternum; (**e**) completion of the web by placing four transverse wires and securing the intersections of the wires; (**f**) covering of the web with a polypropylene mesh. The reconstruction of the soft tissues was then performed using a TRAM flap.

The base of the chest wall reconstruction using this method is a web-like structure inspired by the natural form of a spider web. The fundamental principle is that the inserted web-like framework restores the stability of the chest wall in the area of the defect. The web is constructed from strong, non-resorbable polyester threads (size 1 or 2), placed in such a way that several stable axes are formed. Threads are placed transversally (usually at the site of the resected ribs) and longitudinally (parallel to both lateral resection margins). In contrast to the original “spider web” technique, widely used by Professor T. Horvat and his collaborators at the Central Military Hospital in Bucharest, Romania, we avoid placing pressure on the intercostal nerves via pericostal sutures or inserting wires through bony structures, to reduce postoperative pain. To achieve this, the lateral margin threads are placed in a continuous figure-eight fashion at the cranial margins of the ribs. This configuration creates a stable structure while avoiding nerve compression. The transverse threads are anchored at these two lateral threads rather than using pericostal wires. For larger defects, additional longitudinal threads may be placed within the defect. All wires must be securely tied, ensuring they are not overstretched. This generally leads to a size reduction of the chest wall defect, but care must be taken to avoid bringing the ends of the resected ribs too close together, as this can cause postoperative pain. This thread placement method without direct contact with the intercostal nerves is routinely used in our practice for the closure of any thoracotomy, contributing to the reduction of postoperative pain [6]. Each intersection of the threads is additionally secured with a separate ligature, to ensure tension is distributed in all directions. In cases of resection at the periphery of the chest wall (e.g., first rib, costal arch, or paravertebral region), strong non-resorbable threads are used to replace the first rib or costal arch, or adjacent to the spine, surrounding the adjacent unresected ribs. These threads form a solid framework that allows the web—and later, the mesh—to be securely anchored. Figure 1 illustrates the steps of the “spider web” technique used to close a defect resulting from a large ventral chest wall resection with sternum involvement.

The second layer of the chest wall reconstruction is represented by a synthetic mesh, which facilitates the formation of a fibrous plate through fibroblast colonization. A non-resorbable polypropylene mesh is used in non-infected wound situations. In cases of chest wall reconstruction following resections due to infections, a resorbable polyglycolic acid mesh may be used. In every case, the mesh is tightly secured to the anchoring points of the thread framework with single-knotted sutures, without using pericostal wires. This method of securing the mesh represents the second modification to the original technique. Unlike other techniques that use only a mesh to reconstruct the chest wall, we place the mesh with minimal tension, as the stability of the construct is provided by the “spider web”. Additionally, the mesh can be fixed at certain intersections of the web, further stabilizing the construct and distributing tension in all directions, which prevents tearing at the edges. In cases with small defects or when a synthetic mesh is unavailable, a stable chest wall reconstruction can be achieved using only the web of non-resorbable wires.

### 2.3. Study Design

The data were retrospectively analyzed using a prospectively maintained anonymized database. The evaluation was approved by the Ethics Committee of the University of Lübeck (Reg. No.: 19-102A). Data regarding the indication for chest wall resection, number of ribs resected, the surface of the resulting defect, material used for web formation, type of soft tissue reconstruction, length of hospital stay, technique-specific complications, and parameters related to quality of life were prospectively collected.

### 2.4. Technical and Pulmonary Function Data of the “Spider Web” Thoracic Wall Reconstruction

To analyze the chest wall reconstructions performed, data regarding the size of the reconstructed area, the extent of the resected ribs, the type of material and soft tissue reconstruction used, and the location of the reconstruction were collected. Postoperatively, the 30- and 90-day procedure-specific morbidity and mortality were recorded. In the clinical follow-up, conducted over at least one year, patients were evaluated for chest wall instability, lung herniation, and limitations in upper limb range of motion.

### 2.5. Quality of Life Assessment

At least one year after chest wall reconstruction using the “spider web” technique, the quality of life was assessed using a modified EORTC QLQ-C30 questionnaire [7]. The original EORTC QLQ-C30 questionnaire includes five functional scales and three symptomatic scales. Additionally, it assesses global health status/quality of life (QoL) as well as six individual questions. All scores on the QLQ-C30 range from 0 to 100, with different interpretations depending on the scale. A high score for global health status/QoL indicates a high quality of life, while a high score on a functional scale indicates a high level of functionality. Conversely, a high score on the symptomatic scales indicates significant complaints. This questionnaire was expanded to include pain symptoms, existing pain therapy, and dyspnea symptoms.

Patients received postoperative analgesic management following the principles outlined by the WHO Analgesic Ladder, both during hospitalization and at discharge. In general, a short-term combination of nonsteroidal anti-inflammatory drugs (NSAIDs) and weak opioids (second step) offered optimal pain control within the first weeks after surgery.

### 2.6. Data Analysis

Data were analyzed using GraphPad Prism 6.07 (GraphPad Software, La Jolla, CA, USA). The comparison of pre- and postoperative pulmonary function parameters was conducted using the Wilcoxon Signed Rank Test due to the small sample size. A *p*-value of <0.05 was considered statistically significant.

## 3. Results

### 3.1. Patient Collective and Clinical Course

Over a 5-year period (between 2013 and 2017), a total of 18 chest wall reconstructions using the “spider web” technique were performed on sixteen patients (ten male and six female). Two patients required two chest wall reconstructions on the same site, with the second procedure occurring more than one year after the initial surgery. In the first patient, a metachronous lung carcinoma developed in the right upper lobe following an extended necrotizing chest wall infection due to anastomotic insufficiency after esophagectomy for carcinoma. In the second case, pulmonary metastasis after resection of a chest wall chondrosarcoma required removal.

A total of 11 chest wall resections and reconstructions were performed due to malignancies (liposarcoma, chondrosarcoma, lung cancer, cystosarcoma), four due to metastases (colon cancer and breast cancer), and two for extensive chest wall infections. In one case, resection and “spider-web” reconstruction were indicated due to painful posttraumatic pseudarthrosis. Table 1 summarizes patient characteristics, follow-up period, indication for chest wall resection, hospital length of stay, and procedure-specific complications. The average hospital stay was 24.33 ± 23.01 days, with a postoperative ICU stay of 10.56 ± 23.33 days. In cases involving infection (*n* = 2), the average hospital stay was 48.5 ± 40.74 days, and the postoperative ICU stay was 37.50 ± 42.15 days. For all other cases (*n* = 16), the hospital stay was 18.31 ± 8.57 days, and the postoperative ICU stay was 3 ± 3.92 days. Procedure-specific complications occurred in five cases (27.8%) within the first 30 days postoperatively. One patient developed a seroma, which was evacuated by needle aspiration. Two postoperative hematomas were surgically removed. In one case, necrosis of the TRAM flap donor site occurred, which healed through debridement and vacuum dressing therapy. In the fifth case, a reintervention on the same side to resect a newly diagnosed lung cancer was performed several years after a previous “spider web” chest wall reconstruction for extended infection after esophagectomy. This patient developed an infection of the inserted mesh 9 days after redo reconstruction, leading to mesh explantation. No further procedure-specific complications occurred. The procedure-specific 90-day morbidity and both 30- and 90-day mortality rates were 0%. No local recurrence at the chest wall reconstruction site occurred.

### 3.2. Extent of Thoracic Wall Reconstructions Using “Spider Web” Technique

The extent of chest wall defect reconstructed using the “spider web” technique ranged from two to eight ribs. The average area covered was 195.33 ± 108.72 cm^2^. In 11 cases (61.1%), a non-resorbable polypropylene mesh was used, while in the other seven cases, a resorbable polyglycolic acid mesh was utilized. Resections followed by reconstruction were located in the region in the ventral chest wall with sternal involvement in four cases (22.2%), at the ventral costal arch without sternum involvement in eight cases (44.4%), at the lateral costal arch in four cases (22.2%), and in the paravertebral region in two cases (11.1%). Soft tissue reconstruction was performed using a pectoralis major flap in eight cases, a latissimus dorsi flap in four cases, traverse rectus abdominal muscle (TRAM) flap in two cases, and local muscle without flap mobilization in four cases. Table 2 summarizes the parameters related to chest wall resection and reconstruction: defect area, number of ribs resected, defect location, type of mesh used, and type of soft tissue reconstruction.

### 3.3. Clinical and Pulmonary Functional Course

**Figure 2:**
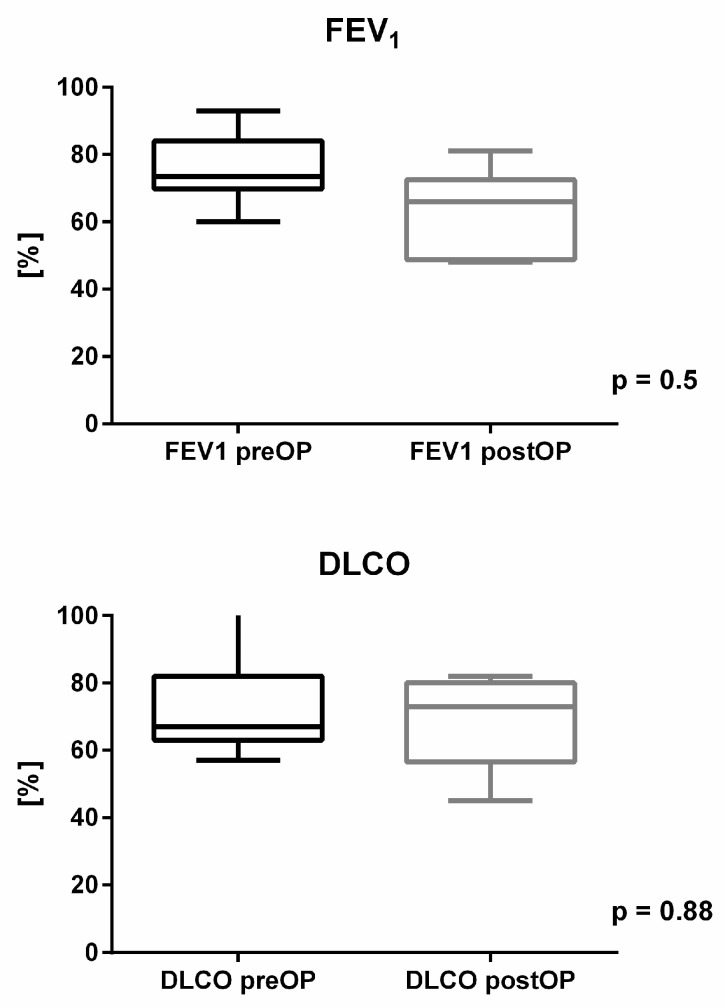
Pulmonary function tests (FEV1 and DLCO) preoperatively and at least one year after chest wall resection and reconstruction using the “spider web” technique (Box-and-Whisker Plots with standard deviation). Neither FEV_1_ nor DLCO demonstrated statistically significant differences at follow-up, with *p*-values of 0.5 and 0.88, respectively.

In the clinical follow-up, all chest wall reconstructions were stable. There were no paradoxical respiratory movements or lung herniations. Additionally, no deficits in the range of motion of the arm or shoulder on the affected side were observed or reported by the patients. All patients were satisfied with the cosmetic outcome. The forced expiratory volume in 1 s (FEV1) and the diffusing capacity of the lungs for carbon monoxide (DLCO) did not show significant reductions in the follow-up assessment, as indicated by *p*-values of 0.5 for FEV_1_ and 0.88 for DLCO (Figure 2). Chronic pain was not identified in our cohort, except for one patient who continued to require analgesic treatment at the one-year follow-up assessment.

### 3.4. Quality of Life

Of the 16 patients, 10 completed the quality-of-life questionnaire (Table 3). The global health status/QoL score was 60 ± 27 points (on a scale from zero to 100, worst to best). At the time of the survey, two patients were undergoing chemotherapy due to metastatic disease originating from their initial malignancy. Functional scales indicated generally good functional status, with physical functioning scored at 69 ± 21 points. Role functioning was rated lower with a score of 58 ± 23 points. Emotional, cognitive, and social functioning revealed higher scores (75 ± 23, 75 ± 29, and 87 ± 21 points, respectively). Symptom scales (on a scale from zero to 100, best to worst) indicated minimal impact from fatigue (42 ± 38 points), sleep disturbances (30 ± 32 points), and appetite loss (23 ± 39 points). Patients reported almost no negative financial impact from the surgical procedure. In the dyspnea survey, none reported shortness of breath at rest. Nine patients (90%) indicated the ability to climb at least one floor of stairs without breathlessness. Half of the patients were able to participate in sports activities. Regarding persistent pain, one patient reported a numeric analog scale (NRS) value greater than four, requiring regular analgesic treatment. Notably, none of the respondents were undergoing the recommended physiotherapy or respiratory therapy at the time of follow-up, suggesting these procedures were not perceived as necessary.

## 4. Discussion

Our experience with the “spider web” technique demonstrates that this is a simple, safe, and cost-effective method for all types of chest wall reconstruction. Moreover, it is particularly well-suited for reconstructions in challenging areas, such as ventral resections with sternum involvement, resections that include the ribs at one of the thoracic apertures, or paravertebral chest wall resections.

The advantage of the “spider web” technique lies in its ability to harness the high stability and flexibility inherent in spider webs, primarily resulting from stress distribution at multiple points. If one of the threads used in reconstruction fails, the overall structure maintains stability until the wires and mesh are fully incorporated. This technique can be employed in both non-infected wound situations, utilizing non-resorbable materials (e.g., strong non-resorbable wires and polypropylene mesh), and in infected wounds, by using resorbable materials (e.g., wires and mesh of polyglycolic acid).

Another key advantage of the “spider web” technique is the use of cost-effective materials that can be easily stored and readily available in most surgical units. Compared to other reconstruction procedures that use elongated polytetrafluoroethylene meshes (ePTFE), polymethylmethacrylate, or metal bars (titanium or steel), the materials used in the “spider web” technique are significantly less expensive.

In contrast to ePTFE meshes, there are currently no data regarding the strength of the reconstruction using the “spider web” technique. However, it must be considered that in case of disruption of one or more fixation sutures, while the ePTFE mesh retains its material properties, the entire construct loses its strength. In contrast, the reconstruction based on the “spider web” technique maintains its stability if a mesh thread is disrupted. One of the major advantages of the “spider web” technique is its ability to be easily performed in anatomically difficult regions where stable reconstruction is challenging, such as defects involving the sternum, resections at the thoracic outlets involving the first rib or costal arch, as well as defects in the paravertebral region. The presented cohort in this study highlights these specific locations, all of which share the characteristic that one margin of the defect is less suitable for strong mesh fixation. This method is particularly suitable for reconstructions at the anterior and lateral chest walls.

As noted by Lampridis et al. in a recent narrative review (2024), the complications following chest wall resections and reconstructions can be categorized into surgical site (procedure-related), respiratory, and other complications. Surgical site complications have a reported incidence of 4–49% and are related to wounds, prostheses, or soft tissue flaps [8].

Wound infection is the major surgical site complication, with an incidence ranging from 2 to 23%, and can be challenging to treat, particularly in cases with concomitant infection of the prosthetic material. Other wound complications include hematoma (2–9.1%), seroma, and dehiscence (1%) [8]. Consistent with these data, in our cohort, hematomas (*n* = 2, 11.1%) and seromas (*n* = 1, 5.6%) occurred at a relatively low frequency. Regarding soft tissue reconstruction, flap failure (3.8–8.8%), bleeding and hematoma (5.9%), and donor site complications have been reported [8]. In our group, wound necrosis at the donor site was encountered in one case (5.6%).

Complications related to prosthetic material are rare with modern materials. Other than soft materials (different types of meshes), rigid materials can be subjected to delayed failure: fracture, displacement, and disconnection between components. For instance, failure of titanium implants after chest wall reconstruction can reach 44.8% on follow-up CT scans, though only 10.3% of patients develop symptoms. The failure risk for titanium implants is higher in anterior reconstructions and the use of more than two bars [8].

Respiratory complications arise from unstable chest wall reconstruction and the loss of lung parenchyma, in cases with associated lung resections. These complications can reach rates as high as 37%, although a correlation between postoperative reductions in lung function and the incidence of pulmonary complications has not been demonstrated [8]. In relation to concerns regarding significant declines in postoperative lung function with the use of soft reconstruction materials, our pulmonary function tests (FEV1 and DLCO) support the clinical observation of optimal functional recovery without significant impairment. In our cohort, FEV1 and DLCO decreased by an average of 17% (75.50 ± 9.72% from predicted value vs. 58.27 ± 19.61% from predicted value) and 1% (70.67 ± 15.77% from predicted value vs. 69.20 ± 14.52% from predicted value), respectively. The difference between the preoperative values and those one year after reconstruction, were not statistically significant. Our results are similar to those published by Leuzzi et al. (2015) [9] on chest wall resections and reconstructions using Vicryl^TM^ (Ethicon, Inc., Johnson & Johnson MedTech, Edinburgh, UK) or Gore-Tex^®^ mesh (W. L. Gore & Associates, Newark, DE, USA) performed in 175 patients. The postoperative reduction in FEV1 (from 87.1 ± 18.9% of predicted to 82.3 ± 23.0% of predicted), forced vital capacity (from 94.1 ± 19.3% of predicted to 82.0 ± 21.6% of predicted) and DLCO (from 15.7 ± 7.4 to 12.1 ± 4.1 mL/min/mmHg) was not statistically significant. Further analysis of their study revealed that the postoperative reduction in pulmonary function was influenced by concurrent lung resection (*p* < 0.001), and the anterior location of the chest wall defect (*p* < 0.026) but not by the resection of more than two ribs [9]. Our clinical data demonstrate that the “spider web” technique offers a stable reconstruction of chest wall defects, as no lung herniations or relevant paradoxical movements were observed. Based on our results and those from other studies using soft materials for chest wall reconstruction, we conclude that no significant deterioration in pulmonary function is to be expected with the “spider web” technique. Interestingly, our findings revealed no increased incidence of post-thoracotomy pain syndrome. At the time of the follow-up assessment, only one patient required analgesics.

In terms of quality of life, although our analysis is based on only ten questionnaires, several interesting observations can be made. When comparing our results with those from the general population aged 50–69 years who did not undergo a chest wall resection, the global health status/QoL showed only a minor reduction of 3.2 to 5.9 points [10]. However, the functional and symptomatic scales revealed postoperative limitations in our patients compared to the general population [10]. This is primarily explained by the tumor condition, which inherently leads to a decline in the functioning scales and has a significant impact on lifestyle and life expectancy [11]. Furthermore, when compared with results from studies on thoracic surgical procedures other than chest wall resections, such as the study by Bendixen et al. on video-assisted thoracic surgery (VATS) versus thoracotomy, the quality of life in our cohort was reduced [12].

A comparison with results from studies analyzing the quality of life after chest wall resections and reconstructions is difficult, as only very few reports were published on this topic. One important study by Salo JTK et al. (2019) assessed the quality of life of 55 oncologic patients following chest wall resections using the QLQ-C30 questionnaire. The global health status/QoL score was 72 points, with functional scale scores ranging from 78 points (physical functioning) to 91 points (social functioning), and symptom scale scores ranging from 2 points (nausea/vomiting) to 23 points (fatigue) [13]. Our results were similar for the functional scales (ranging from 58 points for role functioning to 87 points for social functioning), but poorer for the symptom scales (ranging from 23 points for appetite loss to 42 points for fatigue) and implicitly for global health status/QoL (60 points). These differences are explained by the fact that two of our patients were undergoing chemotherapy at the time of the quality-of-life assessment due to systemic tumor recurrence.

The good results observed in the physical functioning of our patients (69 ± 21 points) correlate with the low score on the dyspnea symptom scale and the good exercise capacity observed, as 90% had no difficulties climbing at least one flight of stairs, and half were able to participate in sport activities.

Chronic pain occurred in only one case (6.25%) in our cohort, showing excellent results compared with rates between 21% and 63% reported by other authors [14,15]. We attribute this finding to the protective attitude towards the intercostal nerves with our suture technique.

Moreover, all patients were satisfied with the cosmetic outcome.

Two main limitations of our study have to be underlined. The first one addresses the small sample size, an aspect that can be attributed to the specific locations of the chest wall reconstructions we chose to highlight. The second limitation is related to the lack of a control arm to compare the “spider-web” technique with other methods. Even though in the distant past we used several other procedures, in the last period, we performed all types of stability reconstructions after chest wall resections exclusively the “spider-web” technique. The decision to abandon all other reconstruction methods was based on our clinical observation that the “spider-web” technique fulfilled all our requirements for chest wall reconstruction (good stability, simple to use, adaptable to all types and sizes of chest wall defects, easily available and cost-effective materials, well incorporated in the tissues). Therefore, our results with the “spider-web” technique were analyzed and discussed using literature data on other techniques, an aspect that limits the strength of the statements formulated. Nonetheless, the good clinical and functional results, the follow-up of at least one year, and the use of a simple technique to reconstruct difficult chest wall defects all strongly support the efficacy of the technique.

The novelty of our study lies in presenting the “spider web” technique as a valuable alternative to other methods for chest wall reconstruction. When compared to the characteristics of the ideal material for chest wall reconstruction proposed by McCormack and Rudolphy, the “spider web” technique offers at least eight out of ten properties (availability, ease of use, adaptability to any size and shape, durability, inertness to body fluids, translucency to x-rays, incorporation by body tissues, malleability to contours) [2,3].

## 5. Conclusions

The “spider web” reconstruction technique for chest wall defects is a simple, safe, and cost-effective method applicable to all types of chest wall reconstructions. It is particularly suitable for reconstructions in challenging areas, such as the sternal region, resections involving one of the thoracic apertures, or resections in the paravertebral region. Moreover, in cases with small defects or if any material is available, the technique can be effectively used to achieve stability without the need for additional mesh coverage. Our study demonstrates that this technique results in minimal morbidity, has no significant impact on postoperative respiratory function, and yields quality-of-life outcomes comparable to other chest wall reconstruction methods.

## Figures and Tables

**Table 1:** Collective description, follow-up duration, indication for chest wall resection and reconstruction, length of hospital stays, duration of ICU treatment, and procedure-specific morbidity.

Sex	male = 10; female = 6
Age (years)	61 ± 9.68
Follow-up duration (days)	756 ± 352.82 [range: 370–1627]
Indication for chest wall resection	
Malignancy	*n* = 11 (61.1%)
Metastases	*n* = 4 (22.2%)
Infection	*n* = 2 (11.1%)
Other	*n* = 1 (5.6%)
Length of hospital stay (days)	
Total	24.33 ± 23.01
Non-infectious disease	18.31 ± 8.57
Infection	48.5 ± 40.74
Intensive care unit	10.56 ± 23.33
Non-infectious disease	3 ± 3.92
Infection	37.5 ± 42.15
Procedure-specific complications	
Seroma	*n* = 1 (5.6%)
Hematoma	*n* = 2 (11.1%)
Wound necrosis at the flap donor site	*n* = 1 (5.6%)

**Table 2:** Parameters related to the chest wall resection and reconstruction.

Defect area (cm^2^)	195.33 ± 108.72
Number of ribs resected	3.47 ± 1.7
Defect location	
- ventral chest wall with sternum involvement	*n* = 4 (22.2%)
- ventral costal arch without sternum involvement	*n* = 8 (44.4%)
- lateral costal arch	*n* = 4 (22.2%)
- paravertebral region	*n* = 2 (11.1%)
Type of mesh	
- Non-resorbable (polypropylene)	*n* = 11
- Resorbable (polyglycolic acid)	*n* = 7
Muscle for soft tissue reconstruction	
- M. pectoralis major	*n* = 8 (44.4%)
- M. latissimus dorsi	*n* = 4 (22.2%)
- TRAM flap	*n* = 2 (11.1%)
- Local muscles	*n* = 4 (22.2%)

**Table 3:** Results of the responses analyzed on EORTC QLQ-C30 questionnaire.

Global health status/QoL	60 ± 27
Functional scales	
- Physical functioning	69 ± 21
- Role functioning	58 ± 23
- Emotional functioning	75 ± 23
- Cognitive functioning	75 ± 29
- Social functioning	87 ± 21
Symptom scales/items	
- Fatigue	42 ± 38
- Insomnia	30 ± 32
- Appetite loss	23 ± 39
- Financial problems	12 ± 27

## Data Availability

The data supporting the findings of this study are available from the corresponding author upon reasonable request. Due to ethical considerations, the data are not publicly accessible.
